# The mortality in infectious inpatients with type 2 diabetes compared with non-diabetic population: Infection in type 2 diabetes

**DOI:** 10.1097/MD.0000000000016025

**Published:** 2019-06-14

**Authors:** Chun-Ming Ma, Fu-Zai Yin

**Affiliations:** a Department of Internal Medicine, Hebei Medical University; b Department of Endocrinology, The First Hospital of Qinhuangdao, No. 258 Wenhua Road, Qinhuangdao, Hebei Province, PR China.

**Keywords:** infection, mortality, type 2 diabetes

## Abstract

The aim of the study was to determine the mortality in infectious inpatients with type 2 diabetes mellitus (T2DM) compared with non-diabetic population.

A retrospective study was conducted on 13,916 infectious inpatients in The First Hospital of Qinhuangdao. Diabetic types were classified using International classification of Diseases-10. Mortality records were collected.

The mortality was higher in patients with T2DM than patients without T2DM (T2DM 4.3% vs non-diabetes 1.7%, *χ*^2^ = 59.560, *P* < .001). In multiple logistic regression analysis, T2DM was an independent risk factor of death in infectious inpatients (OR = 1.539, 95% CI: 1.181∼2.006, *P* = .001). The mortalities between those with T2DM and those without T2DM were stratified by age. The mortalities of patients with T2DM were 0.0% in ∼39 years, 0.0% in 40 to 49 years, 2.7% in 50 to 59 years, 3.1% in 60 to 69 years, 4.1% in 70 to 79 years and 8.7% in 80∼ years groups. The mortalities of patients without T2DM were 0.2% in ∼39 years, 0.1% in 40 to 49 years, 0.6% in 50 to 59 years, 1.0% in 60 to 69 years, 3.1% in 70 to 79 years and 5.9% in 80∼ years groups. T2DM was an independent risk factor of death only in 60 to 69 years groups (OR = 2.323, 95% CI: 1.234∼4.372, *P* = .009).

The increase of mortality appears earlier in patients with T2DM. Infectious inpatients with T2DM are at increased risk of death and brings heavy economic burden to patients, society and government.

## Abstract 2

Supplemental Digital Content is available in the text

## 1 Introduction

Type 2 diabetes mellitus (T2DM) are very common in China. Two national surveys showed that the prevalence of T2DM was around 10%.^[1,2]^ Long-term high blood sugar can cause a series of complications, including cardiovascular disease, stroke, damage to the eyes, chronic kidney disease, foot ulcers, etc. These complications are the major cause of mortality and morbidity in patients with T2DM. Epidemiological studies indicated that with T2DM experienced higher mortality compared to people without T2DM, and that this is mainly through endocrine and cardiovascular cause of death.^[3–8]^

Infection is another major cause of death in patients with T2DM.^[9,10]^ Almost all infections were more common in people with T2DM. Patients with T2DM are at increased risk for lower respiratory tract infection, pneumonia, urinary tract infection, genital and perineal infections, bone and joint infections, and skin, mucous membrane and soft tissue infection.^[11–13]^ People with T2DM are at increased risk of serious infection, and increase infection-related mortality.^[13]^ The data from United Kingdom, Australia and Brazil all indicated that T2DM increased the infection-related mortality.^[13–15]^

The aim of our study was to determine the mortality in infectious inpatients with T2DM compared with non-diabetic population in Qinhuangdao.

## 2 Methods

### 2.1 Subjects

We performed a retrospective study. All subjects were inpatients from the First Hospital of Qinhuangdao between 1 January 2015 and 31 December 2018. The inclusion criteria included the following:

1. all patients were hospitalized due to infections,
2. subjects were men and women over 18 years of age.

The exclusion criteria included the following:

1. subjects with type 1 diabetes,
2. subjects with other specific types diabetes,
3. subjects with no clear type classification,
4. subjects with pre-diabetes,
5. pregnancy and
6. obstetric infection. This study was approved by the ethics committee of the First Hospital of Qinhuangdao.

### 2.2 Classification of infections and diabetic type

Infections were classified using the codes of International classification of Diseases-10 (ICD-10) classifications for hospital admissions. For patients with repeat admissions, the last admission was analyzed. Repeat admissions were recorded.

Diabetic types were classified using ICD-10. The codes of T2DM were included in the study. The codes of type 1 diabetes and other specific types diabetes were excluded. Nonspecific codes and codes of pre-diabetes were also excluded in the study.

### 2.3 Data collection

All data were extracted from the Hospital Information System. Sociodemographic variables were collected and included: age, sex, ethnicity, hospital length of stay (LOS) and hospital cost. Clinical data were collected and included: chronic kidney disease, hypoproteinemia, heart failure, coronary heart disease, stroke & transient ischemic attack(TIA). Intensive care unit (ICU) admissions, septic shock and death were also collected.

### 2.4 Statistical analyses

All analyses were performed using the SPSS 11.5 statistical software (SPSS 11.5 for Windows; SPSS, Inc., Chicago, IL). Numerical variables were reported as mean ± standard deviation and when not normally distributed, they are expressed as medians (interquartile range) and are ln-transformed for analysis. Comparisons were conducted between groups using the *t* test. Categorical data were reported as abnormal subjects (%) and chi-square test was used. Multiple logistic regression models were used for modeling relationships between T2DM and mortality. *P* < .05 was considered statistically significant.

## 3 Results

A total of 16,209 infectious hospital admissions were analyzed. 462 hospital admissions were patients with type 1 diabetes, other specific types diabetes or no clear type classification and 109 hospital admissions were patients with pre-diabetes. The 237 hospital admissions were patients with pregnancy or obstetric infection. These hospital admissions were excluded. The 960 patients with repeat admissions, only the last admissions were included. Eventually, this study enrolled 13,916 patients (7519 males and 6397 females), age 60.1 ± 18.1 years. Supplementary Figure 1 described the process of participant selection.

In these patients, 2236 patients (16.1%) were T2DM. The characteristics of patients with and without T2DM are shown in Table 1. The gender composition between the two groups was similar (*P* > .05). Patients with T2DM were older than patients without T2DM (*P* < .001). The frequencies of CHD, heart failure, CKD, hypoproteinemia, stroke and TIA were higher in patients with T2DM than patients without T2DM (*P* < .001). The levels of hospital LOS and hospital cost were significantly higher in patients with T2DM than patients without T2DM (*P* < .001). The frequencies of repeat admissions, ICU admissions and septic shock were higher in patients with T2DM than patients without T2DM (*P* < .001).

**Table 1:**
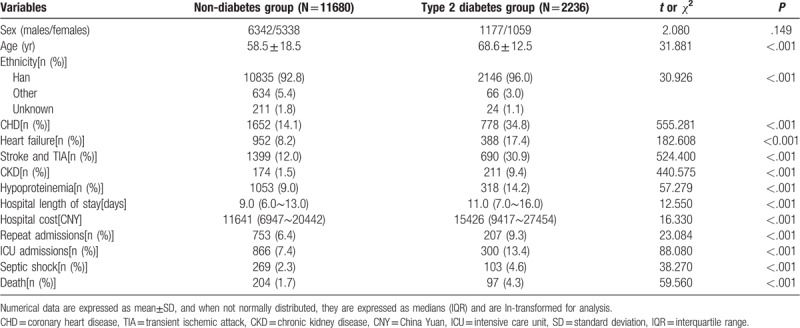
The characteristics of infectious inpatients with and without type 2 diabetes.

The infectious diseases were classified according to the sites of infection. Supplementary Figure 2 shows the 5 most sites of infection. Lower respiratory tract (39.5%), digestive system (19.2%), skin and soft tissue (11.1%), peritoneal cavity (10.9%), urinary and genital tract (5.2%) ranked in the top 5 in non-diabetes group. Lower respiratory tract (51.3%), digestive system (21.1%), urinary and genital tract (8.8%), skin and soft tissue (6.8%), peritoneal cavity (4.1%) ranked in the top 5 in T2DM group.

In these patients, 301 patients (2.2%) died. Supplementary Figure 3 shows the 3 most sites of infection in death. Lower respiratory tract (80.9%), digestive system (7.4%), peritoneal cavity (4.4%) ranked in the top three in non-diabetes group. Lower respiratory tract (81.4%), digestive system (7.2%), peritoneal cavity (2.1%) ranked in the top 3 in T2DM group.

The mortality was higher in patients with T2DM than patients without T2DM (T2DM 4.3% vs non-diabetes 1.7%, *χ*^2^ = 59.560, *P* < .001). In multiple logistic regression analysis, death was considered as the dependent variables with sex, age, ethnicity, T2DM, CHD, heart failure, CKD, hypoproteinemia, stroke and TIA as independent variables. T2DM was an independent risk factor of death in infectious inpatients (OR = 1.539, 95%CI: 1.181∼2.006, *P* = .001). Males, age, heart failure, CKD and hypoproteinemia were also independently associated with death (Table 2). Ethnicity, CHD, stroke and TIA were not included.

**Table 2:**
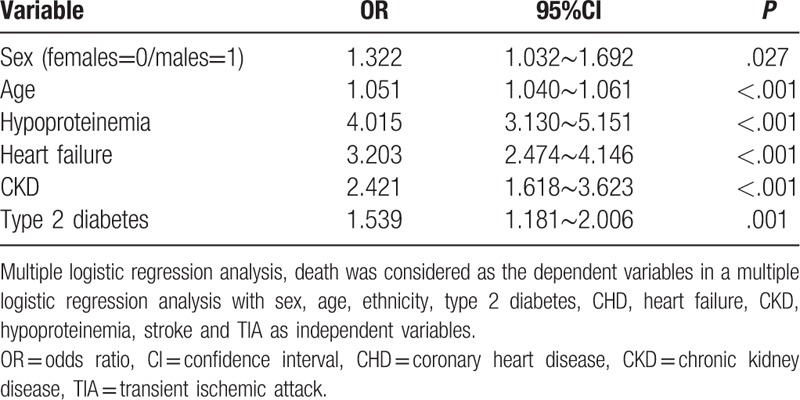
The risk factors of death in infectious patients.

The mortalities between those with T2DM and those without T2DM were stratified by sex and age. The mortalities were higher in patients with T2DM for both genders (*P* < .001) (Table 3). In multiple logistic regression analysis, T2DM was an independent risk factor of death for both genders (males: OR = 1.436, 95%CI: 1.019 to 2.025, *P* = .039 and females: OR= 1.641, 95%CI: 1.077∼2.500, *P* = .021). When infectious inpatients were stratified by age group, patients with T2DM have higher mortalities in 50 to 59 years, 60 to 69 years and 80∼ years groups (*P* < .05) (Table 3). In multiple logistic regression analysis, T2DM was an independent risk factor of death only in 60 to 69 years groups (OR = 2.323, 95%CI: 1.234∼4.372, *P* = .009).

**Table 3:**
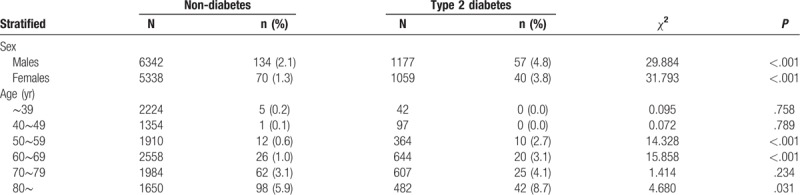
The mortality between infectious patients with and without type 2 diabetes stratified by sex and age.

## 4 Discussion

Our study shows that infectious inpatients with T2DM had longer and more costly hospital stays. The infection could be worse for inpatients with T2DM. The septic shock rate and mortality were higher in infectious inpatients with T2DM. Consistent with previous research,^[16]^ lower respiratory tract was the most commonly affected site in infectious inpatients with T2DM.

Infection constitutes a severe economic burden for global public health systems.^[17–19]^ In our study, the median of hospital LOS was 9 days and the median hospital cost was 11,641 CNY in infectious inpatients. The economic burden is really exacerbated when infectious inpatients accompanied by T2DM. T2DM can prolong the LOS and increase the hospitalization costs in infectious inpatients. Compared with non-diabetic population, the excess LOS for T2DM was 2 days. T2DM contributes to excess expense nearly 4000 CNY in our study. Furthermore, the frequencies of repeat admissions were higher in patients with T2DM. This could lead to a further increase in the economic burden.

Sepsis is when the infection enters the bloodstream and causes inflammation in the body. As a result of these attacks, septic shock can occur and result in multiple organ dysfunction. Septic shock lead to growing ICU admissions.^[20]^ Compared with patients without T2DM, patients with T2DM had higher septic shock rates and ICU admission rates. These results indicate that T2DM associated with the severity of infection in infectious inpatients. Patients with T2DM were more severe than patients without T2DM.

The most serious consequence of disease is death. The mortality was higher in infectious inpatients with T2DM. Lower respiratory tract infection is the leading cause of death in infectious inpatients with T2DM. Patients with T2DM coexisted with multiple concomitant diseases and were on average 10 years older than patients without T2DM. CKD is a common comorbidity in patients with T2DM.^[21]^ CHD and cerebrovascular diseases are the common macrovascular complications, and CHD significantly increased the risk of HF in people with T2DM.^[22,23]^ The risk of infection was higher in patients with these comorbidities being associated with increased mortalities.^[24–27]^ To remove these potential confounders, multifactor analysis was used. After adjustment for potential confounders, we found that a 53.9% increased odds of death associated with T2DM.

In age stratified analyses, the mortalities were similar before the age of 50 years. The mortalities of patients with T2DM started a rapid increase in 50 to 59 years and 60 to 69 years groups. Then, the difference between 2 groups begins to shrink after the age of 70 years. Whether in those with T2DM or in those without T2DM, the mortalities increase with age. However, the increased mortality appeared earlier in those with T2DM.

There are limitations to our study. First, the pathogens of infection included bacteria, virus, fungus, and so on. The pathogens were not analyzed in our study. Second, other confounding factors, such as body mass index, were not evaluated in our study. Third, epidemiologic study proved that the increased mortality is mainly determined by the presence of diabetic complications in T2DM.^[15]^ Long diabetes duration and poor glycemic control is powerfully associated with severity of infection in diabetes.^[28,29]^ These data were not analyzed in our study. Therefore, we cannot analyze the risk of death in T2DM. However, the aim of our study was to determine the mortality in infectious inpatients with T2DM compared with non-diabetic population, not the risk of death in T2DM. These data did not affect the result of our study.

In summary, the increase of mortality appears earlier in patients with T2DM. Infectious inpatients with T2DM are at increased risk of death and brings heavy economic burden to patients, society, and government.

Novelty Statements: The present study showed that the mortality was higher in patients with T2DM than patients without T2DM (T2DM 4.3% vs non-diabetes 1.7%, *χ*^2^ = 59.560, *P* < .001). When the mortalities were stratified by age, the mortalities all increases with age. However, the increased mortality appeared earlier in those with T2DM. Infectious inpatients with T2DM are at increased risk of death and brings heavy economic burden to patients, society and government.

## Author contributions

**Conceptualization:** Fuzai Yin.

**Data curation:** Chun-Ming Ma.

**Writing – original draft:** Chun-Ming Ma.

**Writing – review & editing:** Fuzai Yin.

## Supplementary Material

Supplemental Digital Content
