# Supplementary material for: The mortality in infectious inpatients with type 2 diabetes compared with non-diabetic population: Infection in type 2 diabetes
Source: Medicine (Baltimore). 2019 Jun 14;98(24):e16025. doi: 10.1097/MD.0000000000016025 (PMC6587657; doi:10.1097/MD.0000000000016025)

Supplementary materials

Supplementary Figure 1 Flowchart of participants selection.


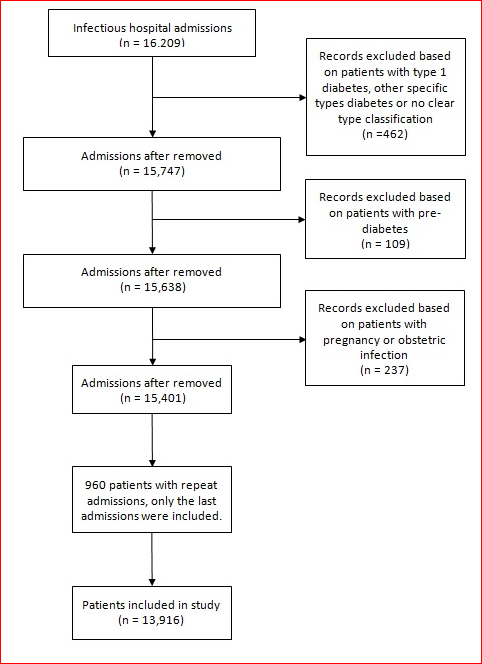


Supplementary Figure 2 The sites of infection in inpatients with and without type 2 diabetes.


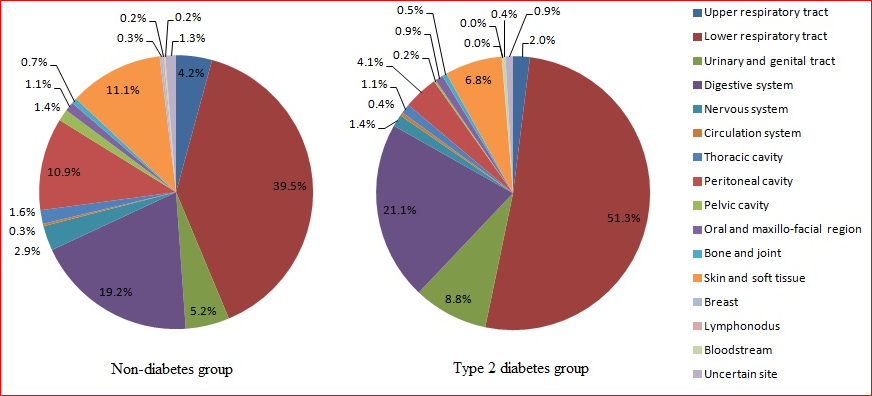


Supplementary Figure 3 The sites of infection in death.


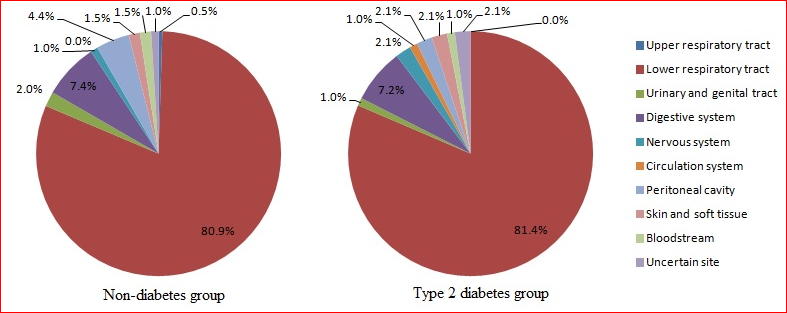

Supplement: Supplemental Digital Content [file medi-98-e16025-s001.doc]
